# *Delftia* sp. LCW*,* a strain isolated from a constructed wetland shows novel properties for dimethylphenol isomers degradation

**DOI:** 10.1186/s12866-018-1255-z

**Published:** 2018-09-06

**Authors:** Mónica A. Vásquez-Piñeros, Paula M. Martínez-Lavanchy, Nico Jehmlich, Dietmar H. Pieper, Carlos A. Rincón, Hauke Harms, Howard Junca, Hermann J. Heipieper

**Affiliations:** 1 Helmholtz Centre for Environmental Research – UFZ, Department of Environmental Biotechnology, Permoserstr. 15, Leipzig, Germany; 2 Technical University of Denmark, Research Data Management – DTU Library, Lyngby, Denmark; 3 Helmholtz Centre for Environmental Research – UFZ, Department of Molecular Systems Biology, Leipzig, Germany; 4 Helmholtz Centre for Infection Research –HZI, Microbial Interaction and Processes Research Group, Braunschweig, Germany; 5 Helmholtz Centre for Environmental Research – UFZ, Department of Environmental Microbiology, Leipzig, Germany; 6 Microbiomas Research Foundation, Bogotá, Colombia

**Keywords:** Xylenols, Biodegradation, Phenol hydroxylase, Toxicity, Constructed wetlands, *Delftia* sp.

## Abstract

**Background:**

Dimethylphenols (DMP) are toxic compounds with high environmental mobility in water and one of the main constituents of effluents from petro- and carbochemical industry. Over the last few decades, the use of constructed wetlands (CW) has been extended from domestic to industrial wastewater treatments, including petro-carbochemical effluents. In these systems, the main role during the transformation and mineralization of organic pollutants is played by microorganisms. Therefore, understanding the bacterial degradation processes of isolated strains from CWs is an important approach to further improvements of biodegradation processes in these treatment systems.

**Results:**

In this study, bacterial isolation from a pilot scale constructed wetland fed with phenols led to the identification of *Delftia* sp. LCW as a DMP degrading strain. The strain was able to use the *o-*xylenols 3,4-DMP and 2,3-DMP as sole carbon and energy sources. In addition, 3,4-DMP provided as a co-substrate had an effect on the transformation of other four DMP isomers. Based on the detection of the genes, proteins, and the inferred phylogenetic relationships of the detected genes with other reported functional proteins, we found that the phenol hydroxylase of *Delftia* sp. LCW is induced by 3,4-DMP and it is responsible for the first oxidation of the aromatic ring of 3,4-, 2,3-, 2,4-, 2,5- and 3,5-DMP. The enzyme may also catalyze both monooxygenation reactions during the degradation of benzene. Proteome data led to the identification of catechol *meta* cleavage pathway enzymes during the growth on *ortho* DMP, and validated that cleavage of the aromatic rings of 2,5- and 3,5-DMPs does not result in mineralization. In addition, the tolerance of the strain to high concentrations of DMP, especially to 3,4-DMP was higher than that of other reported microorganisms from activated sludge treating phenols.

**Conclusions:**

LCW strain was able to degraded complex aromatics compounds. DMPs and benzene are reported for the first time to be degraded by a member of Delftia genus. In addition, LCW degraded DMPs with a first oxidation of the aromatic rings by a phenol hydroxylase, followed by a further *meta* cleavage pathway. The higher resistance to DMP toxicity, the ability to degrade and transform DMP isomers and the origin as a rhizosphere bacterium from wastewater systems, make LCW a suitable candidate to be used in bioremediation of complex DMP mixtures in CWs systems.

**Electronic supplementary material:**

The online version of this article (10.1186/s12866-018-1255-z) contains supplementary material, which is available to authorized users.

## Background

It is well known that the wastewater of coal conversion and coke operation processes contains high concentrations of phenolic compounds, constituting up to 80% of the total chemical oxygen demand (COD) [1]. The phenolic fraction of the this wastewater is mainly represented by phenol, cresols, resorcinols and dimethylphenols (DMP) [2], substances which are considered toxic, carcinogenic, mutagenic and teratogenic [3]. The DMPs, have shown to be even more persistent and toxic than other phenolic compounds [4] and they are often released to the environment in higher concentration than the permissible limits established [1, 5].

Biological treatment of phenols and their derivatives has been the main approach for their removal [6, 7]. Biodegradation of the six DMP isomers, in pure or mixed cultures, often involves different *Pseudomonas* species [8–10]. Degradation of 2,6-DMP has only been reported by *Mycobacterium* sp. strain DM1 [11]. Studies aiming at the removal of DMP in a mixture with other substituted phenols in laboratory-scale sludge units have also been performed [6, 12]. Recently, constructed wetlands (CWs) have been investigated for their potential to remove coke oven wastewater [13]. Promisingly, Schultze-Nobre et al., reported efficient removal of a mixture of three DMP-isomers (2,6-, 3,4- and 3,5-DMP) in a laboratory scale CW [14].

Complex physicochemical and biological processes in CWs include the interaction of plants, microorganism and pollutants. The main role in the transformation and mineralization of organic pollutants is played by microorganisms present in the rhizosphere, an environment suitable for highly efficient transformations of complex contaminants [15, 16]. There is, however, little knowledge about the bacterial removal of DMP in CWs and detailed microbiological and molecular biological studies are needed to reveal the biological activities towards DMP as a basis for better management of CWs.

In this work, we aimed to isolate strains from a constructed wetland treating phenolic wastewater in order to characterize the ability to degrade DMP isomers, the toxicity tolerance toward the isomers and to reveal the genomic and proteomic mechanisms supporting the degradation. The first results indicated that *Delftia* sp. LCW is a versatile bacterium with novel properties to degrade DMP isomers and it could be used to enhance phenolic compounds removal in CWs.

## Results

### DMP-degrading bacterium, growth and co-metabolism assays

From the isolation trials, LCW was the strain recovered with capabilities to use DMP as sole carbon and energy source. According to its 16S rRNA gene sequence, the isolate is a member of the genus *Delftia*. The 16S rRNA gene is identical to that of *Delftia* sp. strain SM-1 (JN001163) and differs by only 1–2 bases from that of various *Delftia acidovorans* isolates (e.g. *D. acidovorans* SPH-1, CP000884 and *D. acidovorans* NBRC 14950, AB680719; 99.9% sequence identity) but by 7–8 bases from that of *Delftia tsuruhatensis* and *Delftia lacustris* strains, including the respective type strains (*D. lacustris* 332 EU888308; *D. tsuruhatensis* T7, AB075017).

**Fig. 1:**
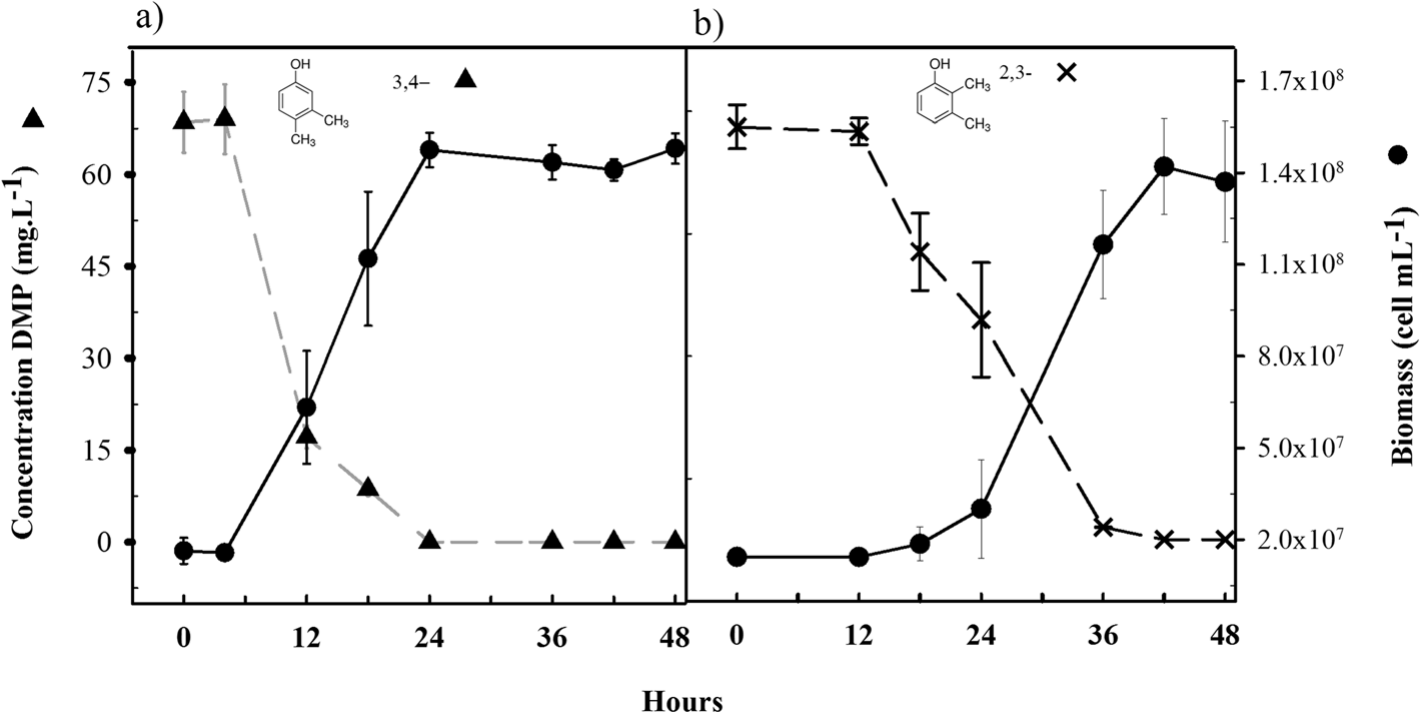
Growth (circle) of *Delftia* sp. LCW and degradation of **a** 3,4-DMP (triangle) and **b** 2,3-DMP (cross) as sole carbon and energy source. Bars represent ±SD

The strain was able to use 2,3-DMP and 3,4-DMP as sole carbon and energy source (Fig. 1a and b). Complete depletion of the isomers was observed and both substrates yielded similar bacterial biomass (Table 1).

**Table 1:** Yield coefficients and bacterial biomass for *Delftia* sp. LCW grown on DMP-isomers. The overall initial concentration of the DMP in all treatments was 70 mg L^− 1^

DMP	Maximal Biomass (cell mL ^− 1^)	Yield (mg dry weight mg^−1^ C-DMP)
3,4-	1.48 × 10^8^ ± 5.66 × 10^6^	0.73 ± 0.04
2,3-	1.42 × 10^8^ ± 1.56 × 10^8^	0.69 ± 0.62
3,4- + 2,3-	1.49 × 10^8^ ± 1.27 × 10^7^	0.73 ± 0.07
3,4- + 3,5-	7.88 × 10^7^ ± 4.10 × 10^6^	0.28 ± 0.04
3,4- + 2,5	8.61 × 10^7^ ± 1.01 × 10^7^	0.39 ± 0.02
3,4- + 2,4	6.48 × 10^7^ ± 1.54 × 10^7^	0.35 ± 0.02
3,4- + 2,6	6.24 × 10^7^ ± 1.82 × 10^6^	0.30 ± 0.66

**Fig. 2:**
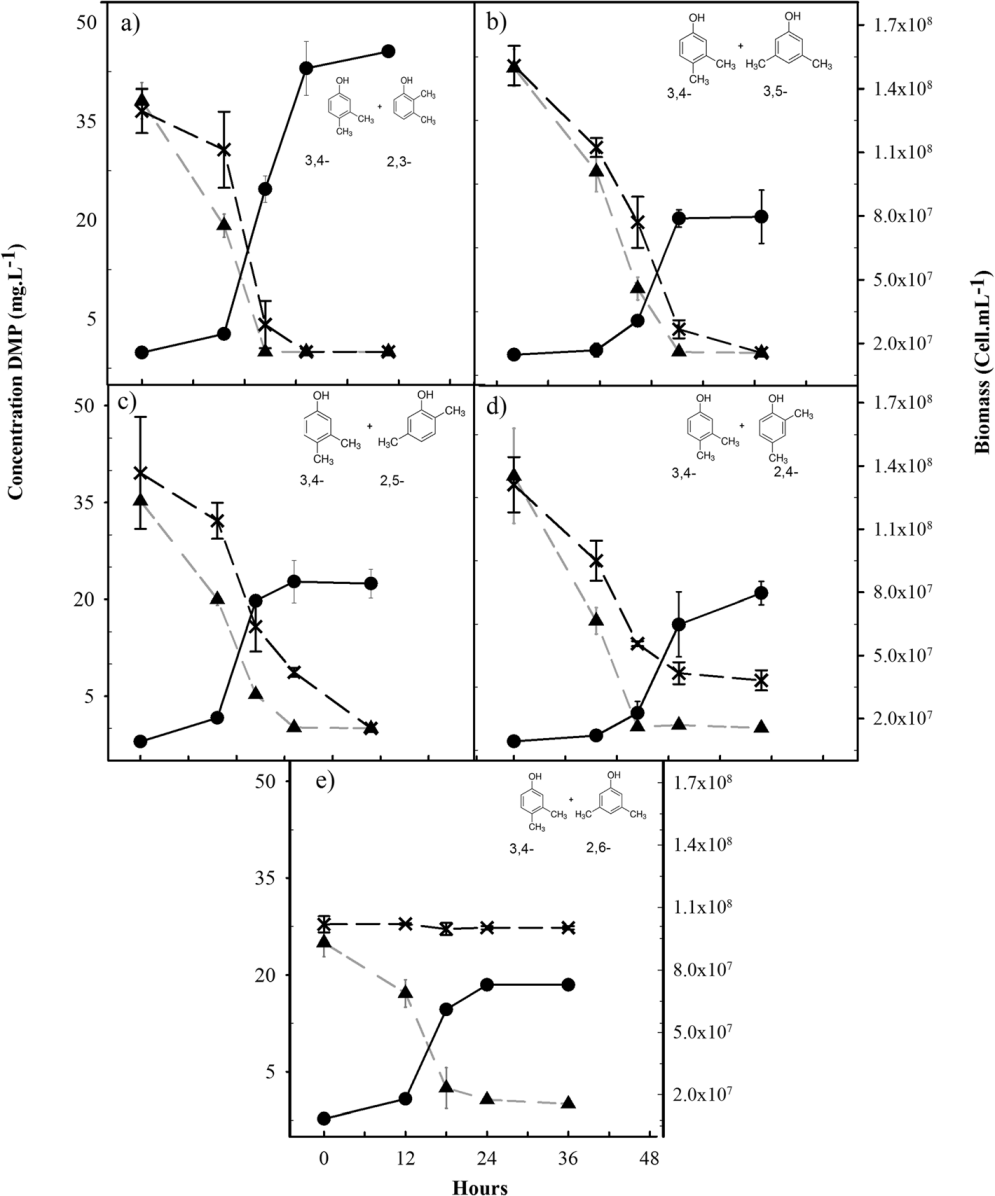
Growth (circle) of *Delftia* sp. LCW and degradation of the isomeric mixtures. **a** 3,4-DMP (triangle) and 2,3-DMP (cross), **b** 3,4-DMP (triangle) with 3,5-DMP (cross), **c** 3,4-DMP (triangle) with 2,5-DMP (cross), **d** 3,4-DMP (triangle) with 2,4-DMP (cross), and **e** 3,4-DMP (triangle) with 2,6-DMP (cross). Bars represent ±SD

In mixtures of 3,4- and 2,3-DMP the latter isomer sharply decreased the lag phase (Fig. 2a). *Delftia* sp*.* LCW was not able to grow with the other four DMP isomers. However, in the presence of 3,4-DMP, the isomers 2,5- and 3,5-DMP were completely transformed (Fig. 2b and c), whereas partial transformation of 2,4-DMP (Fig. 2d) and no transformation of 2,6-DMP were observed (Fig. 2e). The mixture of 3,4- and 2,3- DMP gave rise to a similar yield to the one obtained with 3,4-DMP alone (Table 1). Meanwhile, mixtures with 3,4- plus 3,5-, 2,5- or 2,4-DMP yielded approx. half of the bacterial yield (Table 1). In addition, bacterial biomass was significantly higher in the mixture of 3,4- and 2,3- DMP than the biomass of the other DMP mixtures (*p* < 0.001, Additional file 1: Table A). Therefore, it was evident that none cell growth was giving by 3,5-, 2,5- and 2,4-DMP. No abiotic losses were detected in any DMP isomer solutions, proving that the decrease in DMP concentrations was due to microbial activity (data not shown).

### Detection of aerobic catabolic genes in *Delftia* sp. LCW

**Fig. 3:**
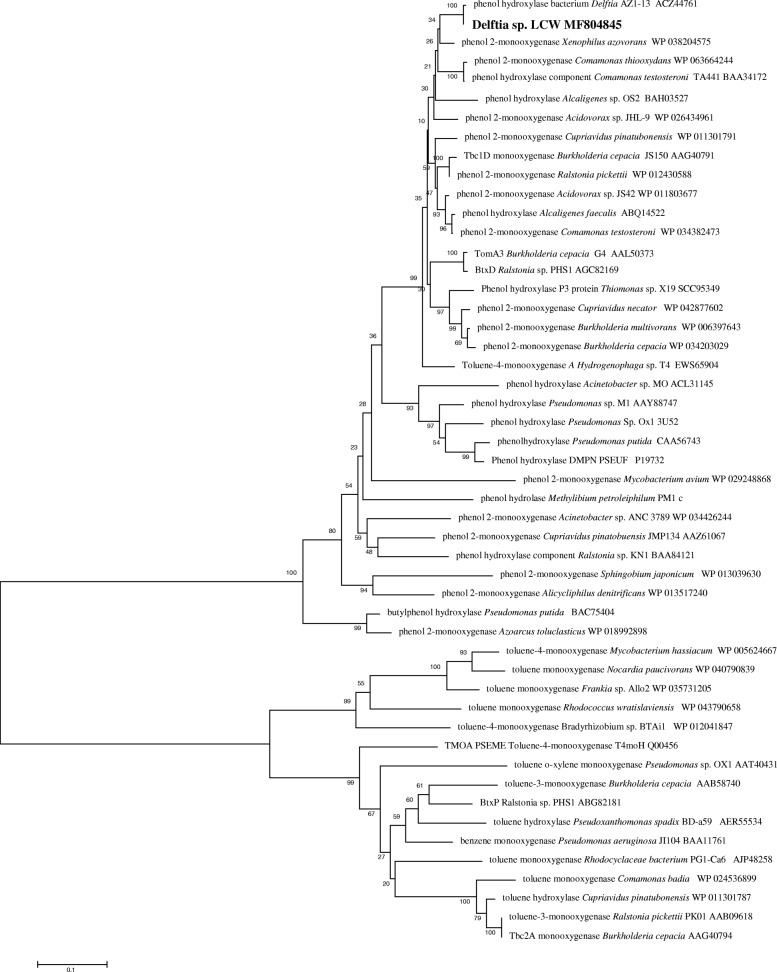
Phylogenetical analysis of the phenol monooxygenase of *Delftia* sp. LCW and related proteins, using neighbor-joining method

Among the different primer sets targeting catabolic genes tested with *Delftia* sp*.* LCW, amplification was only observed with phenol hydroxylase gene primers. The deduced protein sequence had a length of 214 amino-acids and the most closely related identified protein was the phenol hydroxylase large subunit from *Delftia* sp. AZ1–13 (ACZ44761). The protein clustered with phenol hydroxylases of *Comamonas testosteroni* TA441 (BAA34172) and other members of the family Comamonacea, as well as some few toluene monooxygenases of *Burkholderia cepacea* (stain JS150-AAG40791 and strain G4-AAL50373) (Fig. 3). It was not possible to detect amplification of other BTEX catabolic genes with the set of primers used.

### Growth of *Delftia* sp LCW. on other aromatics

*D. acidovorans* grew on 30 mg L^− 1^ benzene to a biomass of 7.44 × 10^7^ ± 1.46 × 10 cell x mL within 48 h. Growth of the strain on other BTEX compounds was not observed.

### Proteomic analysis

The label-free shotgun proteomics approach allowed the identification of 1814 proteins (Additional file 2). First, an unsupervised clustering approach (Principal Component Analysis, PCA) based on the different treatments, revealed clear segregation by sample treatment. The variances among the treatment groups showed that replicate analyses are clustering together. The PCA analysis also showed a greater segregation between the proteome profile of 3,4-DMP and 2,3-DMP among the four treatments (Additional file 3). The proteins that were significantly different between 3,4- and 2,3-DMP, were functionally assigned according to KO (KEEG Orthology), which characterizes gene functions in order to infer high level protein functions of the organism.. The main functional categories of the proteins that differed between 3,4- and 2,3-DMP treatments can be found in Additional file 4.

**Fig. 4:**
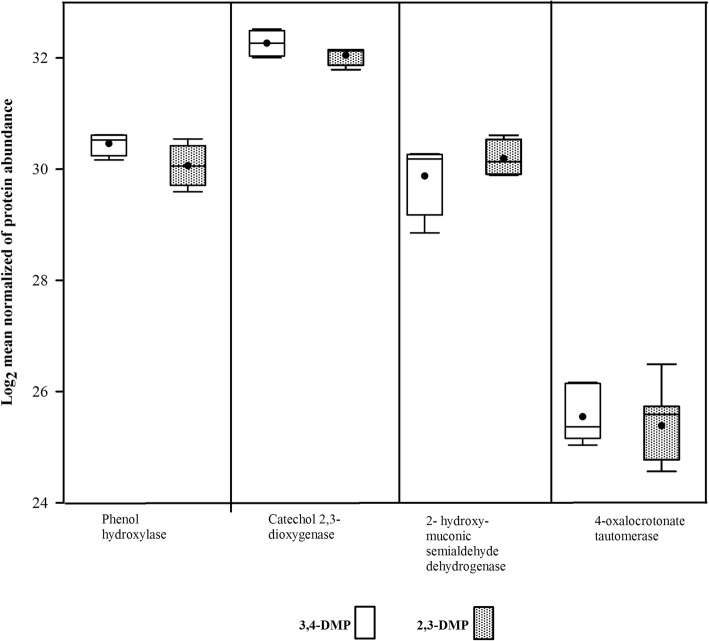
Abundance of representative proteins involved in DMP degradation with 3,4- and 2,3-DMP as singles isomers. Bars indicate mean ± SD. No statistical differences were found (*p* > 0.05)

In total, five proteins related with aromatic degradation pathways could be identified (Table 2). Phenol hydroxylase, catechol 2,3-dioxygenase, 2-hydroxymuconic-semialdehyde dehydrogenase and 4-oxalocronate tautomerase were identified in all treatments. Protocatechuate 4,5-dioxygenase was identified only in 2,3-DMP grown cells. In Fig. 4, the protein abundance of related DMP proteins present in 2,3- and 3,4-DMP as singles isomers are indicated. There were not significant differences of the abundances of the proteins between for 2,3- and 3,4-DMP treatments, for any of the proteins related with aromatic degradation (phenol hydroxylase, *p* = 0.160; catechol 2,3-dioxygenase, *p* = 0.227, 2-hydroxymuconic semialdehyde dehydrogenase, *p* = 0.488 and 4-oxalocronate tautomerase *p* = 0.227) (Additional file 1: Table B).

**Table 2:** Identified proteins of *Delftia* sp. involved in phenol catabolic pathways

Treatment	Entry	Protein names	Gene names	Organism	Peptide length (bp)	Inferred DMP -catabolic pathway
3,4-, 2,3- and mixtures	D1LCK1	Phenol hydroxylase large subunit		Bacterium AZ1–13	186	Catechol *meta* and *ortho* cleavage
3,4- 2,3- and mixtures	Q60GE8	Catechol 2,3-dioxygenase	ORF7NH	*Delftia acidovorans*	314	Catechol *meta* cleavage
2,3-	A9C0K2	Protocatechuate 4,5-dioxygenase	Daci_4445	*Delftia acidovorans* (strain DSM 14801 / SPH-1)	289	Protocatechuate *meta* cleavage
3,4-, 2,3- and mixtures	Q8KRR9	2-hydroxymuconic semialdehyde dehydrogenase	nahI	*Pseudomonas fluorescens*	486	Catechol *meta* cleavage
3,4-, 2,3- and mixtures	A0A1C7L505	4-oxalocrotonate tautomerase	ACM14_28930	*Delftia* sp. JD2	138	Catechol *meta* cleavage

### Toxicity of DMP-isomers to *Delftia* sp. LCW

**Fig. 5:**
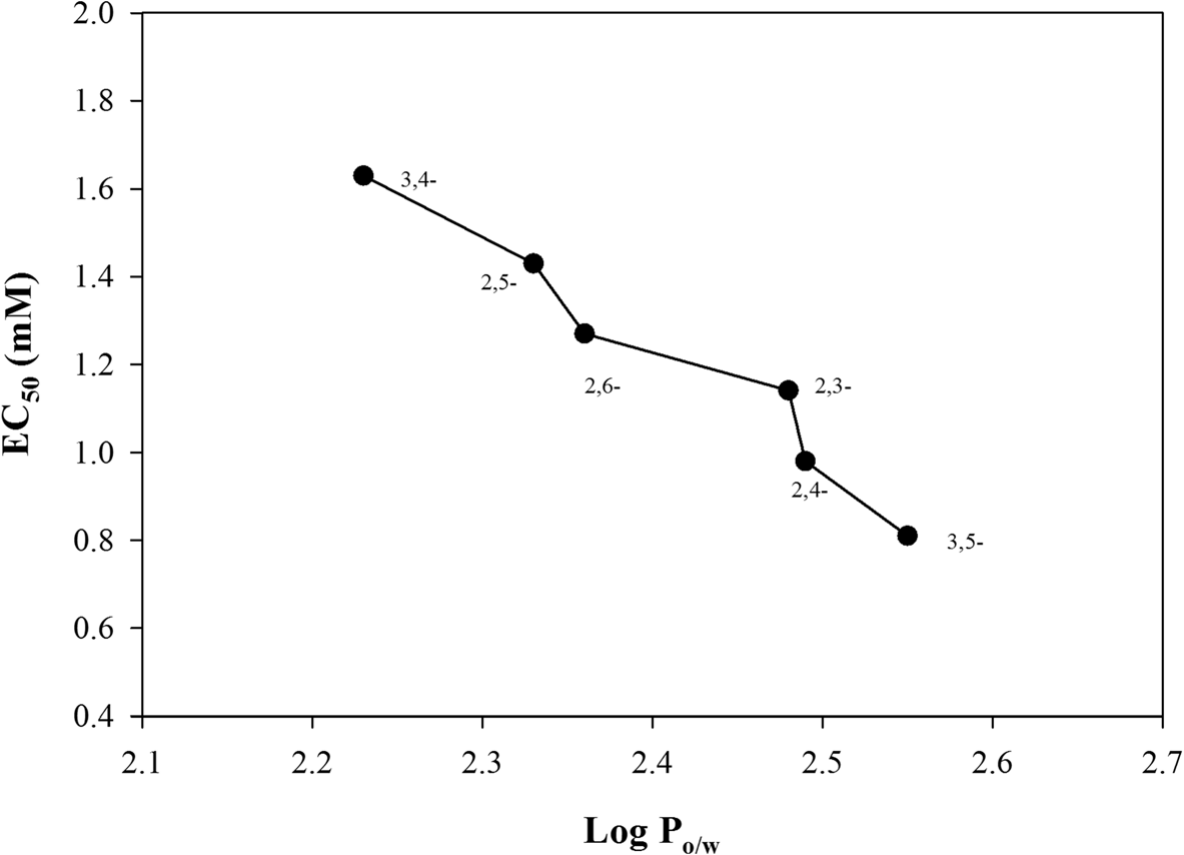
Correlation between EC_50_ (half maximal effective concentration) and Log P_ow_ (partition coefficient octanol/water) for the six DMP-isomers

Pearson correlation coefficient between EC_50_ and the log P_ow_ showed a negative correlation for all DMPs-isomers (correlation coefficient − 0.978 and *p*-value =0.0007). Hence, 3,5-DMP (represented by the highest log P_ow_) had the lowest EC_50_ value, followed by 2,4-, 2,3-, 2,6-, 2, 5- and 3,4-DMP (Fig. 5).

## Discussion

Based on the best performance to degrade DMP among the obtained isolates, the strain named as *Delftia* sp*.* LCW was selected. This isolate proved to be a close relative of the type strain *Delftia* sp. SM-1 and several strains of *Delftia acidovorans*. Strains of *D. acidovorans*, formerly known as *Comamonas acidovorans* [17], have been reported as degraders of 4-nitrobenzoate [18], 2,4-dichlorophenoxyacetic acid (2,4-D) and 4-chloro-2-methylphenoxyacetic acid (MPCA) [19]. Other isolates from the genus *Delftia* are able to degrade a wide range of phenoxyalkanoic acid herbicides, chlorinated phenols [20–22], sodium dodecyl sulfate [23], phenanthrene [24] and taurocholate [25]. Another family related bacterium, *Comamonas testosteroni* CNB-2 has been also reported to degrade 4-chlorophenol (4-CP), phenol and methylphenols [26, 27]. However, to our knowledge this is the first report of a *Delftia* strain able to degrade DMP and benzene.

*Delftia* sp. LCW was able to grow on the *ortho*-DMP isomers as a sole carbon and energy source. A similar growth profile has previously reported for *Cupriavidus pinatobuensis* JMP 134 [28], *Pseudomonas* CF600 [10, 29], *Pseudomonas aeruginosa* T1 [30] and *Comamonas testosteroni* JH5 [26].

In general, DMP biodegradation have been reported for all isomers, mainly through four different pathways, 1) monooxygenation of the aromatic ring to form dimethylcatechols followed by extradiol ring-cleavage as reported for 2,3- and 3,4-DMP degradation [28, 31], 2) oxidation of the methylsubstituent to form methyl-substituted gentisates followed by degradation through a gentisate degradative pathway as reported for 2,5-, and 3,5-DMP degradation [32, 33]; 3) oxidation of both methylsubstituents to form 4-hydroxyisophthalate followed by hydroxylation and degradation via a protocatechuate pathway as reported for 2,4-DMP degradation [34, 35] or 4) two successive monooxygenation of the aromatic ring 2,6-dimethylhydroquinone and 2,6-dimethyl-3-hydroxyhydroquinone as reported for 2,6-DMP degradation [11]. Proteome analysis showed the expression of a catechol *meta* cleavage pathway by *Delftia* sp. LCW in the presence of 3,4-DMP and 2,3-DMP including a catechol 2,3-dioxygenase, a 2-hydroxymuconic semialdehyde dehydrogenase and a 4-oxalocronate tautomerase. Catechols will be formed by a phenol monooxygenase, where the gene encoding the alpha-subunit (P3 subunit) was also detected in the genome through PCR, in order to produce the corresponding dimethylcatechol. A respective pathway for the degradation of 3,4-DMP was previously observed in *Pseudomonas* sp. CF600 and *Pseudomonas putida*. P35X [10, 31, 36].

Although catechol *meta* cleavage enzymes were detected in the presence of 2,3-DMP, an additional enzyme was identified when the isomer was provided as sole carbon source. This enzyme was the protocatechuate 4,5-dioxygenase (A9C0K2). Other proteins belonging to the protocatechuate pathway were not detected in the proteomic analysis. The protocatechuate pathway has been described only in 2,4-DMP degradation by *Pseudomonas putida* NCBIMB 9866 where it is formed through the subsequent oxidations both methyl group of 2,4-xylenol to form 4-hydroxyisophthalate and hydroxylation to protocatechuate [35]. Our results indicate that *Delftia* sp. LCW produced enzymes of the protocatechuate pathway in the presence of 2,3-DMP provided as a sole carbon source. However, this is probably due to fortuitous induction. Since proteins were identified by searching against UNIPROT database for genera related to *Delftia* or reported DMP-degrading bacteria, only the anticipated proteins listed could be identified.

When a mixture of 3,4- and 2,3-DMP was provided, there was an evident shorter lag phase for the degradation of 2,3-DMP. Fostered degradation of DMP in mixtures with other aromatic compounds was previously observed in a sequencing batch reactor, where degradation kinetics of 3,4-DMP were improved in the presence of 4-nitrophenol (4-NP) [12]. Likewise, experiments with *C. testosteroni* had shown that the addition of phenol increased cell growth and shortened the time necessary for 4-Clorophenol (CP) degradation [27]. Similar experiments with *Pseudomonas putida* XQ23 showed acceleration of 2,3-DMP degradation in the presence of DMP mixtures [37]. Those finding, in addition to higher abundance of *meta* pathway proteins in the mixture of the *ortho* DMP, suggested that the presence of 3,4-DMP in the mixture induced the expression of the catechol *meta* cleavage pathway and fostered the degradation 2,3-DMP.

Strain LCW was not able to grow on any of the other DMP isomers as a single substrate. Methylated phenols with methyl groups in ring position five (2,5 and 3,5-DMP), have been reported to be degraded by bacteria via gentisate pathway metabolism [32]. Proteins related to gentisate pathways were not detected in *Delftia* sp. LCW. Members of *Delftia* genus typically encode gentisate pathway. However, the appropriate methylhydroxylase has not been found in *Delftia* genomes, according to the search in PATRIC database. LCW strain transformed 2,4-, 2,5- and 3,5-DMP in the presence of 3,4-DMP. Mixtures of 3,4- and 2,3- DMP led to higher microbial yields than mixtures with 3,4- plus 3,5-, 2,5- or 2,4-DMP, showing the ability of *Delftia* sp. LCW to use only 3,4- and 2,3-DMP as a carbon source, and to remove 3,5-, 2,5- and 2,4-DMP by co-metabolic transformation. Similar co-metabolic transformation of 2,4-, 3,4- and 3,5-DMP has been reported for different strains of *Pseudomonas* [9] and *C. testosteroni* CPW301 [27]. It was also reported that *C. testosteroni* CPW301 was able to partially degrade 2,5- and 3,5- DMP by co-metabolism when growing with 4-CP and 4-MP [26]. In the case of *Delftia* sp. LCW, 3,4-DMP induced the enzymatic activity that allowed the transformation of 2,4-; 2,5- and 3,5-DMP and fostered 2,3-DMP degradation.

Screening of catabolic genes using degenerated primers and PCR led to the detection of the fragment of the phenol hydroxylase large subunit [38, 39]. The phenol hydroxylases comprise related family of enzymes capable to hydroxylate mainly phenols and their methyl-substituted derivatives to the corresponding catechols [40, 41]. Some phenol hydroxylases families have showed also the ability to transform toluene or benzene [42]. Phylogenetical relationships of phenol hydroxylase from LCW, showed that the closest protein is phenol hydroxylases from *Delftia* AZ1–13 (ACZ44761), which phenotype (L6) was a representative in a lab-scale reactor treating phenols [43]. Phylogenetical clustering of the phenol hydroxylase of LCW revealed similarities with the phenol hydroxylase *Comamonas testosteroni* TA441 (BAA34172) [44]. It was also similar to other strains with reported genes for catechol *meta* cleavage pathway e.g. *Cupriavidus pinatubonensis* [28, 45]. Another related phenol hydroxylase of LCW, belonging to *Pseudomonas* sp. M1 (ABM96259) with high similarities to the *dmpKLMNOP* hydroxylase gen of CF600 [46] .

In general, the catabolic pathway of 3,4- and 2,3- DMP by *Delftia* sp. LCW is similar to the one previously reported for *Cupriavidus pinatobuensis* JMP 134 [28], *Pseudomonas* sp*.* CF600 and *Pseudomonas putida* P35X [10, 29], *Pseudomonas aeruginosa* T1 [30] and *Comamonas testosteroni* JH5 [26]. Furthermore, phenol hydroxylases are reported to be of broad substrate specificity and to transform, phenol, 2-methylphenols, 2,4 and 3,4-DMP, *o*-cresol, *m*-cresol [28, 40, 47, 48], and are able to transform 2,4-DMP and 2,5-DMP [47]. Based on our findings, it is assumed that phenol hydroxylase of *Delftia* sp. LCW is not only responsible for the initial degradation of 2,3- and 3,4-DMP, but when induced by the presence of 3,4-DMP, it would be also able to transform other non-growth DMP isomers.

*Delftia* sp. LCW was also able to use benzene as growth substrate. One known pathway for the degradation of benzene involves two successive monooxygenations that are catalyzed by different soluble diiron monooxygenases [45]. Nevertheless, some phenol monooxygenases may catalyze both successive monooxygenations [40]. This characteristic was found in *Pseudomonas* sp. M1, where the multicomponent hydroxylase *phc* gene was responsible for the initial oxidation of phenol or benzene [46], this subunit is indeed similar to the one found in LCW. In addition, the phenol hydroxylase gene large subunit sequence found in LCW showed similarities with the toluene/benzene/chlorobenzene-monooxygenase (tbc1D) from *Burkholderia* sp. strain JS150 (AAG40791). Considering the detection of this gene sequence, the corresponding protein expressed and the phylogenetic similarities with other well-characterized functional proteins, we can propose that this phenol hydroxylase detected in *Delftia* sp. LCW may be responsible of catalyzing both monooxygenation reactions during the degradation of benzene.

Previous toxicity assays performed in bacterial strains such as *Vibrio fischeri*, showed highest toxicity of 3,4-DMP compared to other DMP-isomers, cresols, resorcinol and other dihydroxyphenols [4, 49, 50]. 3,4- and 2,3-DMP from activated sludge treating phenols-like wastewater had been reported to be toxic for eukaryotic multicellular organisms such as the ecotoxicological indicators *Daphnia magna* and *Thamnocephalus platyurus* [4]. In bacteria, there are cultures of *Pseudomonas* strains where the highest concentration of 3,4-DMP supporting growth was in the range of 2.5 mM [51], i.e. similar to the current findings with *Delftia* sp. LCW. The toxicity tests of the different DMP isomers on *Delftia* sp. LCW showed that 3,5-DMP was the most toxic isomer followed by a decreasingly toxic series in the following order: 2,4-, 2,3-, 2,6-, 2,5- and 3,4-DMP (Fig. 5).

Considering that only 3,4-DMP contributed to 8.5% for the phenol toxicity in ash heap water from an oil shale industry [52] and given the fact that this isomer can impair the performance of biological treatment processes of phenolic wastewater [4], the tolerance and degradation of 3,4-DMP by *Delftia* sp. LCW, together with its role for co-metabolic transformation of other DMP isomers support the proposal of LCW strain as a promising microbial tool component on further developments of wastewater treatment technologies of phenolic compounds.

## Conclusions

LCW strain was able to degraded complex aromatics compounds. DMPs and benzene are reported for the first time to be degraded by a member of Delftia genus. In this strain, the isomer 3,4-DMP acted as an inducer of phenol hydroxylase enzyme, that is responsible for a first oxidation of the DMPs, and it is followed by a further *meta* cleavage pathway for *ortho*-DMPs isomers. Such induction also led to the transformation of 2,4-, 2,5- and 3,5-DMPs. The higher resistance to DMP toxicity, and the understanding of degradation pathways for DMPs by LCW strain, as well as its origin as a rhizosphere bacterium from CWs, make of *Delftia* sp. LCW a suitable candidate to be used in bioremediation of coke-coal contaminated sites and is important to establish technological alternatives for wastewater treatment for coal-coke industry effluents.

## Methods

### Isolation and identification of DMP degrading bacteria

Bacterial strains were isolated by selective enrichment from a pilot-scale horizontal sub-surface CW fed with groundwater with benzene, phenols and *m*-cresols. In order to select strains with potential ability to degrade DMP for subsequent assays, bacterial growth on each of the six single isomers of DMP as a sole carbon source and energy was determined. The 16S rRNA gene from the only isolate showing growth on DMP isomers and called strain LCW, was amplified by PCR using universal bacterial primers 27F and 1492R [53]. Sanger sequencing of the purified PCR product was obtained (GATC Biotech AG, Cologne, Germany) and the 16S rRNA gene sequence was compared to public databases using RDP [54] to identify closest reference sequences The 16S rRNA gene sequence from strain LCW is available in KY643688k with accession number KY643688 [55].

### DMP degradation and co-metabolism assays

In order to evaluate degradation and co-metabolic capabilities of the isolated, pure cultures of the strain were transferred from plates to liquid LB medium and incubated overnight at 30 °C with agitation. Then, cells were harvested by centrifugation, washed three times with phosphate buffer (50 mM of NaH_2_PO_4_, pH 7.0) and re-suspended in 50 mL of liquid M9 [56] (turbidity of ~ 0.05 at 620 nm) with the corresponding carbon source (as described below). First, the selected isolate was grown on 3,4-DMP and 2,3-DMP separately with a concentration of 70 mg L^− 1^ each. In addition, co-metabolic transformation by the strain was tested using each isomer (2,3-, 2,4-, 2,5-, 2,6- and 3,5-DMP) mixed with 3,4-DMP in concentrations of 35 mg L^− 1^ per isomer (i.e. 70 mg L^− 1^ DMP in total). Each treatment was set up in triplicates. Samples were taken from each culture every 4 and 12 h to determine cell number and DMP concentration, until the stationary phase was reached. Growth was followed by cell counting using a Coulter Counter® (Beckman Coulter Inc) and the software Multisizer 3 Version 3.51® (Beckman Coulter Inc.). The concentration of DMP in the cultures was measured in cell free supernatants by HPLC (Promicence, Shimadzu) equipped with a UV detector and a 2.7 μm by 3.0 × 150 mm poroshell 120 EC-C18 column (Agilent Technologies, USA). For the separation of the DMP isomers, the mobile phase A consisted of formic acid (0.1%) and phase B consisted of acetonitrile (100%), using the following gradients over a total run time of 45 min: 20% B to 40% B with a flow rate of 0.2 mL min^− 1^ [14]. For each treatment, a flask prepared under the same conditions without bacterial inoculum was set up in order to determine abiotic losses. In order to compare differences in degradation, the approximate bacterial yield for each mixture was calculated according to the following formula:

$$ Yield=\frac{Increase\ in\ bacterial\ biomass\kern0.5em \left( mg\  dry\  biomass.{mL}^{-1}\right)}{C\  from\  DMP\  consumed\ \left( mg.{mL}^{-1}\right)} $$

$$ Increase\ in\ bacterial\ biomass=\kern0.5em \frac{\left({C}_{tf}\  cell.{mL}^{-1}-{C}_{t0}\  cell.{mL}^{-1}\right)\times 3\  mg.\kern0.5em {mL}^{-1\ast }\ }{1x{10}^7\  cell.{mL}^{-1}}\ \mathrm{where}, $$

$$ {C}_{tf}=\mathrm{number}\ \mathrm{of}\ \mathrm{cells}\ \mathrm{per}\ \mathrm{mL}\ \mathrm{at}\ \mathrm{the}\ \mathrm{end}\ \mathrm{of}\ \mathrm{the}\ \mathrm{expontial}\ \mathrm{phase} $$

$$ {C}_{t0}=\mathrm{number}\ \mathrm{of}\ \mathrm{cells}\ \mathrm{per}\ \mathrm{mL}\ \mathrm{at}\ \mathrm{time}\ \mathrm{cero} $$

* A theoretical value of 3 mg mL^− 1^ of dry mass equivalent to 1 × 10^7^ cell mL^− 1^ for Gram negative rod bacteria was calculated [57]

*C from DMP consumed* = (*DMP*_*t*0_ *mg*. *mL*^−1^ − *DMP*_*tf*_ *mg*. *mL*^−1^) × 0.787* where,

$$ {DMP}_{t0}= Concentration\ of\ the\ corresponding\  DMP\  isomer\  at\  time\ cero $$

$$ {DMP}_{tf}=\mathrm{Concentration}\ \mathrm{of}\ \mathrm{the}\ \mathrm{DMP}\ \mathrm{isomer}\ \mathrm{at}\ \mathrm{the}\ \mathrm{end}\ \mathrm{of}\ \mathrm{the}\ \mathrm{expontial}\ \mathrm{phase} $$

*1 M of DMP contains 78.7% of carbon

### Growth of *Delftia* sp. LCW on other aromatic compounds

In order to investigate the spectrum of aromatics that *Delftia* sp*.* LCW was able to degrade*,* the strain was incubated with BTEX compounds. Initially, the strain was grown on LB and incubated overnight at 30 °C with agitation. Cells were harvested by centrifugation, washed three times with phosphate buffer [50 mM of NaH_2_PO_4_, (pH 7.0)] and re-suspended in 50 mL of M9 medium (turbidity of 0.05 at 620 nm) and the selected sole carbon source at a concentration of 30 mg L^−1^. The growth tests were performed in duplicate. In addition, flasks prepared under the same conditions without carbon sources were tested as a negative control. Aliquots were taken for turbidity measurements at 620 nm every 12 h for 2 weeks.

### Detection and identification of aerobic catabolic genes in *Delftia* sp. LCW

*Delftia* sp*.* LCW was assessed for the presence of catabolic genes potentially involved in aerobic degradation of aromatic compounds. Detection of monooxygenases [38, 39, 58], intradiol catechol dioxygenases [59] and extradiol catechol dioxygenases [39, 60] in the genome of strain LCW was performed by PCR using previously described degenerate primers (Additional file 5). The obtained PCR products were purified and sequenced (GATC Biotech AG, Cologne, Germany). The obtained nucleic acid sequences were translated and compared to entries in public databases [61]. The only detected gene sequence from this PCR specific target survey is available in GeneBank with accession number MF804845. Protein translation was obtained through EMBL-EBI translation tool service [62], where, frame 3 from the positive strand was chosen. PATRIC genome database [63] was used in order to check the detected gene in other related genus members.

### Proteomics analysis

Protein extraction, LC-MS measurements and protein identification were performed as described previously by Lünsmann et al., [64]. Briefly, for protein extraction, cells were grown on 3,4- DMP and 2,3-DMP individually and on mixtures of 3,4- with 2,3- and 3,4- with 3,5-DMP as described before. Bacterial cells were harvested in the middle of the exponential phase. Lysis was achieved by ultrasonication and proteins were recovered by denaturation and solubilization with urea buffer. The protein lysates were applied on a polyacrylamide gel electrophoresis. Gel pieces were cut and the proteins were lysed using trypsin overnight. The resulting peptide lysates were separated by liquid chromatography hyphenated with mass spectrophotometry (LC-MS). For the identification of proteins, the acquired LC-MS data were searched against the public database UniprotKB using the closest related genera, i.e. *Delftia*, *Pseudomonas*, *Ralstonia*, *Comamonas* and bacterium AZ1–13, as inferred from the sequenced Phenol hydroxylase fragment. LC-MS spectra were searched using the Proteome Discoverer (Thermo Fisher Scientific, v1.4, San Jose, CA, USA). Search settings were: Sequest HT search engine, trypsin (full specific), MS tolerance 10 ppm, MS/MS tolerance 0.02 Da, two missed cleavage sides, dynamic modifications oxidation (Met), static modifications carbamidomethylation (Cys). Only peptides that passed the FDR thresholds of < 1% FDR q-value (Percolator) and rank 1 peptides were considered for further analysis. Label-free quantification was done using the Top-3 peptide area for approach. These linear area values were log_10_-transformed, median normalized and furthermore missing value imputation were performed. Principal component analysis (PCA) was performed using InfernoRDN (version 1.6044.35184 July 19, 2016). Hypothesis significance tests were performed with Prostar [65] using the threshold of the log fold change (FC) of 1 and the threshold of –log (*p*-value) of 2 for protein abundances. The proteins significantly differing between incubations with the 3,4- and 2,3-DMP were functionally assigned with the annotation tool KEGG BlastKOALA [66].

### Toxicity test

*Delftia* sp*.* LCW was grown in mineral medium M9 with 4 g L^− 1^ of disodium succinate as carbon and energy source in 50-mL flasks. Once the cultures reached the exponential phase of growth, DMP isomers were added separately at different concentrations (one flask per concentration) ranging from 45 to 350 mg L^− 1^. No DMP isomers were added to the control culture. For each flask, the OD_620mn_ was measured every hour until the growth stopped in one of the cultures flasks. To determine the toxicity of each compound, specific growth rate μ (h^− 1^), was calculated as follows [67]:

$$ \upmu\ \left[{h}^{-1}\right]=\frac{{\mathit{\ln}}_{x_{t2}}-{\mathit{\ln}}_{x_{t1}}}{t_2-{t}_1} $$ where,

*μ* [*h*^−1^] = Growth rate

x_t1_ = OD_620_ at time t_1_

x_t2_ = OD_620_ at time t_2_

t_1_ = one hour after toxin was added [h]

t_2_ = final time when the growth stopped [h]

Inhibited growth (%) was defined as the percentages of the growth rates μ (h^−1^) of toxified cultures divided by the growth rate of the control culture. EC_50_ was calculated for each isomer by interpolation of the concentration resulting in 50% growth inhibition. Log P_ow_ values were obtained from Toxnet Database [68].

### Statistical analyses

In order to indicate significant differences among values of maximal bacterial biomass in the cometabolic assay and protein abundances of selected proteins in proteomics data, normal distributions were tested with Shapiro Wilk and further parametric or not parametric test were performed accordingly. In addition, Pearson correlation was performed to correlate EC_50_ and Log P_ow_ values for all isomers. Analyses were performed using SigmaPlot Version 13.0 (®Systat Sofware, Inc.). *P*-values and specific statistical tests are described in Additional file

## Additional files

Additional file 1: **Table A.** *p*-values for the cometabolic assay, and **Table B.** p-values of paired t-tests for the comparison between 2,3- and 3,4-DMP. (DOCX 16 kb)

Additional file 2: **Table S2.** Proteome raw data for DMP treatments (XLSX 22980 kb)

Additional file 3: **Figure S1.** Principal component Analysis of the proteomic profile of strain LCW for the four DMP treatments. (PNG 37 kb)

Additional file 4: **Figure S2.** Proteins functional category of *Delftia* sp. LCW for the proteins with significant differences between 3,4- and 2,3- DMP isomers (proteins with p-value < 0.001 for FC were selected for the analysis). (TIF 100 kb)

Additional file 5: **Table S1.** Primers used for targeting catabolic genes in the genome of *Delftia* sp. LCW (DOCX 34 kb)

## Abbreviations

BTEX: Benzene, toluene, ethylbenzene and xylenes
CL: Chlorophenol
COD: Chemical oxygen demand
CW(s): Constructed Wetland(s)
DMP: Dimethylphenols
EC_50_: Half maximal effective concentration
EMBL-EBI: European Molecular Biology Laboratory-The European Bioinformatics Institute
FC: Fold change
FDR: False discovery rate
HPLC: High performance liquid chromatography
KEGG: Kyoto encyclopedia of genes and genomes
KO: KEGG orthology
LB: Lysogeny broth
LC-MS: Liquid chromatography-mass spectrometry
LCW: *Delftia* sp. strain LCW
Log Pow: Partition coefficient octanol/water
MP: Methylphenol
NP: Nitrophenol
PATRIC: Pathosystems Resource Integration Center
PCA: Principal components analysis
RDP: The Ribosomal Database Project
UniprotKB: UniProt Knowledgebase
