# Supplementary material for: Delftia sp. LCW, a strain isolated from a constructed wetland shows novel properties for dimethylphenol isomers degradation
Source: BMC Microbiol. 2018 Sep 6;18:108. doi: 10.1186/s12866-018-1255-z (PMC6127914; doi:10.1186/s12866-018-1255-z)
Supplement: Supplementary file 1 — Table A. p-values for the cometabolic assay, and Table B. p-values of paired t-tests for the comparison between 2,3- and 3,4-DMP. (DOCX 16 kb) [file 12866_2018_1255_MOESM1_ESM.docx]

Table A. p-values for the cometabolic assay. One way ANOVA for the comparison of bacterial biomass (cell·mL-1) among the DMP groups obtained p-value= < 0.001. Further all pairwise multiple comparison Holm-Sidak method was applied to see differences between each group. Overall significance level =0.05

| **Group comparison** | | **p-value** | **Significance** |
| --- | --- | --- | --- |
| 3,4- & 2,3- vs | 3,4- & 2,4- | <0.001 | *** |
|  | 3,4- & 2,5- | <0.001 | *** |
|  | 3,4- & 2,6- | <0.001 | *** |
|  | 3,4- & 3,5- | <0.001 | *** |
| 3,4- & 2,5- vs | 3,4- & 2,4- | 0.147 |  |
|  | 3,4- & 2,6- | <0.001 | *** |
|  | 3,4- % 3,5- | <0.001 | *** |
| 3,4- & 3,5- vs | 3,4- & 2,4- | 0.005 | ** |
|  | 3,4 & 2,6- | <0.001 | *** |
| 3,4 & 2,4- vs | 3,4- & 2,6- | 0.218 |  |

Table B. p-values of paired t-tests for the comparison between 2,3- and 3,4-DMP. The test was performed for each protein involved in DMPs’ degradation pathways. Overall significance level =0.05. No significant differences were found in any of the selected proteins (p>0.01).

| **Comparison** | **p- value (two tail)** |
| --- | --- |
| 3,4- & 2,3- (all proteins) | 0.227 |
| Phenol hydroxylase (3,4- vs 2,3-) | 0.16 |
| Catechol 2,3 dioxygenase (3,4- vs 2,3-) | 0.488 |
| 2-hydroxymuconic semialdehyde (3,4- vs 2,3-) | 0.299 |
| 4-oxalocronate tautomerase (3,4- vs 2,3-) | 0.227 |
