# Supplementary figures and images for: Delftia sp. LCW, a strain isolated from a constructed wetland shows novel properties for dimethylphenol isomers degradation
Source: BMC Microbiol. 2018 Sep 6;18:108. doi: 10.1186/s12866-018-1255-z (PMC6127914; doi:10.1186/s12866-018-1255-z)

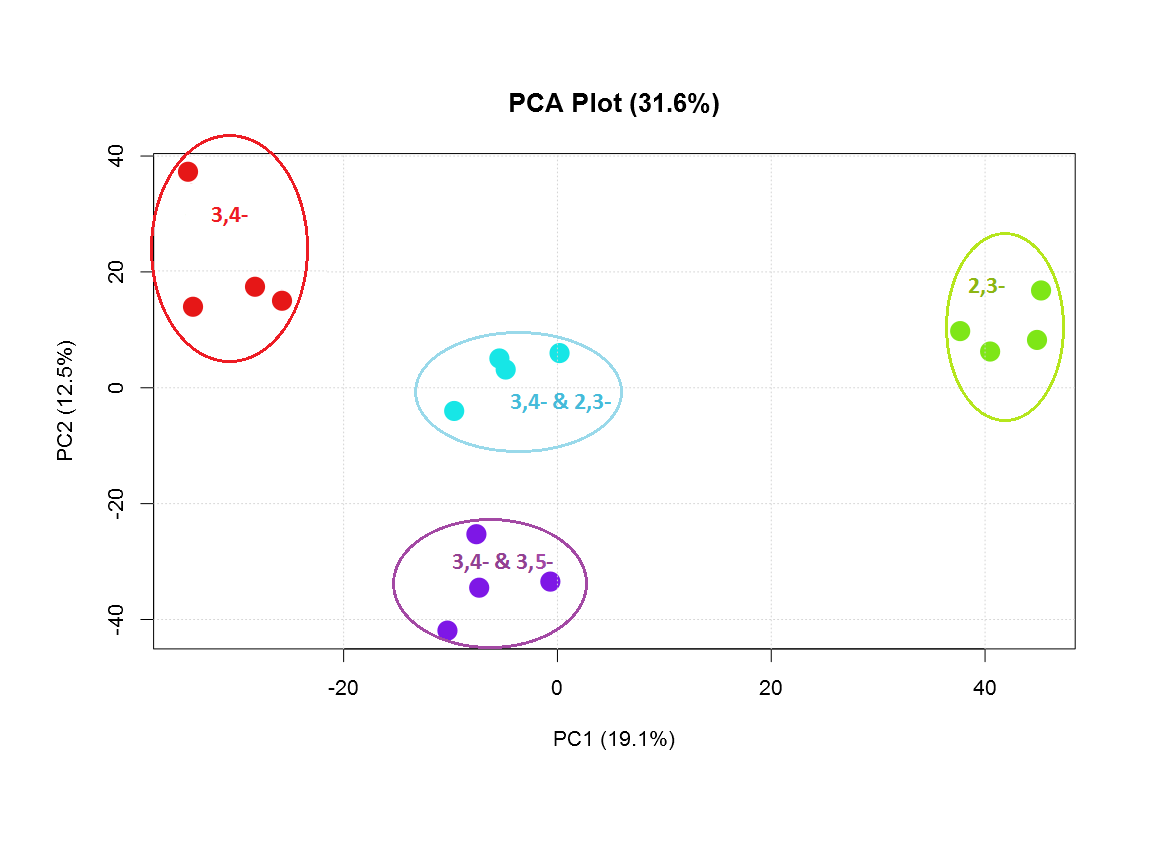

Supplement: Supplementary file 3 — Figure S1. Principal component Analysis of the proteomic profile of strain LCW for the four DMP treatments. (PNG 37 kb) [file 12866_2018_1255_MOESM3_ESM.png]

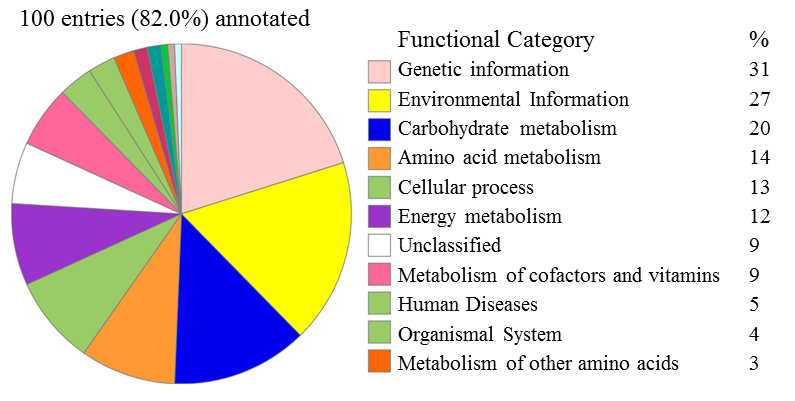

Supplement: Supplementary file 4 — Figure S2. Proteins functional category of Delftia sp. LCW for the proteins with significant differences between 3,4- and 2,3- DMP isomers (proteins with p-value < 0.001 for FC were selected for the analysis). (TIF 100 kb) [file 12866_2018_1255_MOESM4_ESM.tif]
