# Supplementary material for: Delftia sp. LCW, a strain isolated from a constructed wetland shows novel properties for dimethylphenol isomers degradation
Source: BMC Microbiol. 2018 Sep 6;18:108. doi: 10.1186/s12866-018-1255-z (PMC6127914; doi:10.1186/s12866-018-1255-z)
Supplement: Supplementary file 5 — Table S1. Primers used for targeting catabolic genes in the genome of Delftia sp. LCW (DOCX 34 kb) [file 12866_2018_1255_MOESM5_ESM.docx]

**Table S1.** Primers used for targeting catabolic genes in the genome of *Delftia* sp. LCW.

| Primers | Target gene | Sequence (5′-3′) | Size (pb) | | Reference |  |
| --- | --- | --- | --- | --- | --- | --- |
| **EXDO-D** | C23O subfamily I.2.C extradiol dioxygenases | F= AAYCCBGABCCNTGGCCNGA  R1=GTYTSVCCNCGBGTVADVCCRTGRCG  R2=ATRTCVAKVGADGTRTCGSTCATG | 380 | [[1](#_ENREF_1)] | | |
| **EXDO-L** | C23O of Proteobacteria related to TodE | F= GACCAGGGSWTVGGYCACTA  R= TTRTGNCCCCAGATGCTGAT | 380 | [[1](#_ENREF_1)] | | |
| **Tbu-E** | TbuE (U20258) of subfamily I.2.C of catechol extradiol dioxygenases | F= CTGGATCATGCCCTGTTGATG  R=CCACAGCTTGTCTTCACTCCA | 505 | [[2](#_ENREF_2)] | | |
| **TmoA** | Subfamily 2 of α-subunits of hydroxylase component of multi-component mono-oxygenases | F=CGAAACCGGCTT(C/T)ACCAA(C/T)ATG  R=ACCGGGATATTT(C/T)TCTTC(C/G)AGCCA | 505 | [[2](#_ENREF_2)] | | |
| **PHE (H)** | α-subunit of phenol-methylphenol monooxygenases | F=GAYCCBTTYCGY HTRACCATGGA  R= GGCARCATGTAR TCCWKCATCA | 701 | [[3](#_ENREF_3)] | | |
| **C12O** | Catechol C120 | F=GCCAACGTCGACGTCTGGCA  R=GCCAACGTCGACGTCTGGCA |  | [[4](#_ENREF_4)] | | |
| **TodC** | Subfamilies D.1.B+D.1.C+D.2.A+D.2.B+D.2.C of a-subunits of Type D iron–sulfur multi-component aromatic dioxygenases | F=CAGTGCCGCCA(C/T)CGTGG(C/T)ATG  R=GCCACTTCCATG(C/T)CC(A/G)CCCCA | 510 | [[2](#_ENREF_2)] | | |
| **TBMD** | Subfamily 1 of a-subunits of hydroxylase component of multi-component mono-oxygenases | F=GCCTGACCATGGATGC(C/G)TACTGG  R=CGCCAGAACCACTTGTC(A/G)(A/G)TCCA | 640 | [[2](#_ENREF_2)] | | |

# References

1. Brennerova MV, Josefiova J, Brenner V, Pieper DH, Junca H: Metagenomics reveals diversity and abundance of meta-cleavage pathways in microbial communities from soil highly contaminated with jet fuel under air-sparging bioremediation. *Environ Microbiol* 2009, 11(9):2216-2227.

2. Hendrickx B, Junca H, Vosahlova J, Lindner A, Ruegg I, Bucheli-Witschel M, Faber F, Egli T, Mau M, Schlomann M *et al*: Alternative primer sets for PCR detection of genotypes involved in bacterial aerobic BTEX degradation: distribution of the genes in BTEX degrading isolates and in subsurface soils of a BTEX contaminated industrial site. *J Microbiol Methods* 2006, 64(2):250-265.

3. Martinez-Lavanchy PM, Chen Z, Lunsmann V, Marin-Cevada V, Vilchez-Vargas R, Pieper DH, Reiche N, Kappelmeyer U, Imparato V, Junca H *et al*: Microbial Toluene Removal in Hypoxic Model Constructed Wetlands Occurs Predominantly via the Ring Monooxygenation Pathway. *Appl Environ Microbiol* 2015, 81(18):6241-6252.

4. Sei K, Asano K, Tateishi N, Mori K, Ike M, Fujita M: Design of PCR primers and gene probes for the general detection of bacterial populations capable of degrading aromatic compounds via catechol cleavage pathways. *J Biosci Bioeng* 1999, 88(5):542-550.
